# The Facial Osteoplasty for Polyostotic Fibrous Dysplasia in a Patient With McCune-Albright Syndrome: A Case Report

**DOI:** 10.7759/cureus.48526

**Published:** 2023-11-08

**Authors:** Paula Vitória Bido Gellen, Gustavo Paiva Custódio, Alef Vieira Galvão, Bruna Estrozi, Alessandro Rocha de Lellis

**Affiliations:** 1 Traumatology and Maxillofacial Surgery Department, Hospital das Clínicas, Goias Federal University, Goiânia, BRA; 2 Pathology Department, Hospital das Clínicas, Goias Federal University, Goiânia, BRA

**Keywords:** oral and maxillofacial surgeons, facial bones, osteotomy, fibrous bone dysplasia, mccune-albright syndrome

## Abstract

Fibrous dysplasia is a developmental anomaly that affects bone maturation and remodeling, generating replacement of medullary and cortical bone by a disorganized and immature fibro-osseous matrix, which makes the sufferer susceptible to bone pain, skeletal deformities, and pathological fractures. This is a condition that, when associated with cutaneous hyperpigmentation and endocrinological disorders, forms the classic triad of McCune-Albright syndrome, a rare multisystemic pathology formed by postzygotic somatic mutations of the GNAS gene. Fibrous dysplasia can even cause loss of vision, hearing, or difficulty breathing. The severity of these manifestations is associated with the type of treatment, which can be conservative or surgical. The surgical approach is adapted to each particularity and it aims mainly to resolve functional restrictions or correct aesthetic deformities through bone plasty. The present work aims to report the case of a McCune-Albright syndrome patient with deforming craniofacial fibrous dysplasia that triggers respiratory compromise. After clinical and tomographic evaluation, it was decided to remove and reshape the bone affected in the maxilla through the Weber-Ferguson approach and the mandible through the modified Newman approach. The case progressed satisfactorily, with an improvement in the respiratory condition and a reduction in facial asymmetry.

## Introduction

McCune-Albright syndrome is a rare multisystemic pathology, with a prevalence between 1:100,000 and 1:1,000,000, with a gradual, painless onset, slow growth rate and classically composed by the triad of polyostotic fibrous dysplasia, café-au-lait skin pigmentation and precocious puberty [1-3].

The etiology involves the inherent activation of adenyl cyclase and overproduction of cyclic adenosine monophosphate, responsible for the postzygotic somatic mutation of the GNAS gene on chromosome 20q13-13.29, responsible for encoding the stimulatory G protein subunit [1]. Is believed that this syndrome is secondary to postzygotic mutations attributing characteristics sustained by the absence of vertical transmission of the disease and skin and bone lesions that rarely cross the midline [1].

The clinical phenotype of McCune-Albright syndrome varies depending on the location and time of mutation in embryological development, the number of mutant cells and affected organs [1]. Therefore, it is valid to add that the main complications in the craniofacial skeleton are associated with the expansion of fibrous dysplasia, a component of a rare group of bone dysplasias, characterized by the production of fibro-osseous tissue and loss of normal medullary characteristics, which results in osteolytic lesions, bone pain, skeletal deformities and increased chances of pathological fractures [2-5].

Given the possible complications, the diagnosis of fibrous dysplasia in the craniofacial region must be agile and efficient [6]. This is constructed through the association of clinical, imaging and histopathological examination [5].

Fibrous dysplasia can be managed conservatively through observation and follow-up, through drug therapy using bisphosphonates or denosumab and, finally, through surgical therapy through plasty. The invasive approach aims to control pain, reduce bone deformities, prevent the occurrence of pathological fractures and is normally performed after puberty, due to the possibility of remission of the fibro-osseous lesion [2,3,5].

The present study aims to report the case of a McCune-Albright syndrome patient with an important dysplastic manifestation in the craniofacial region with deforming repercussions on the face and respiratory function. The management of the case involved a detailed analysis of the medical history, with records of three previous osteoplasties, clinical and tomographic examination to define the surgical approach, which involved the removal of excess fibro-osseous lesion and local plasty in the maxilla through the access of Weber-Ferguson and mandible via modified Newman approach. The patient presented satisfactory evolution in the post-surgery period, with functional and aesthetic reestablishment and improvement in quality of life.

## Case presentation

A female patient, 23 years old, feoderm, diabetic and with McCune-Albright syndrome with a history of eight fractures in the lower limbs and maxillary osteoplasty in December 2018, March 2020 and March 2021 due to craniofacial fibrous dysplasia, attended the Oral and Maxillofacial Surgery and Traumatology with complaints of respiratory difficulty and deforming expansion of the face. On clinical examination, significant facial asymmetry combined with bilateral maxillary growth was observed, with greater prominence on the right side, associated with displacement of the nasal floor and lateral wing of the nose with partial obstruction and grade I tooth mobility of the maxillary dental elements. Furthermore, the increase was also verified in the region of the parasymphysis and mandibular body on the right side with grade III mobility in elements 44 and 45.

**Figure 1:**
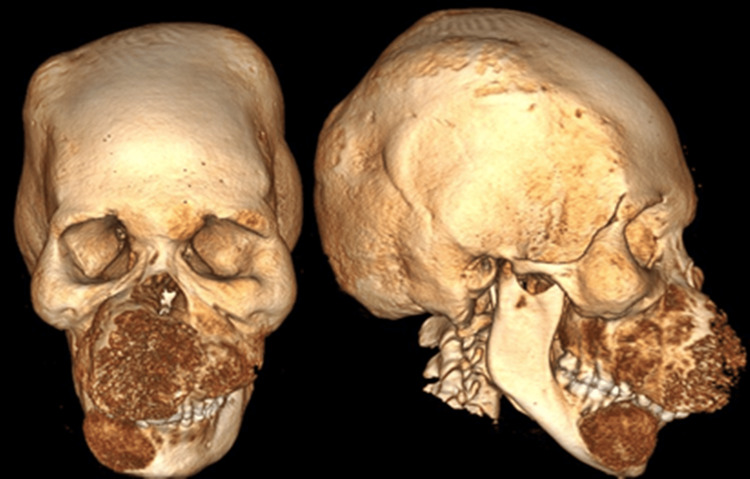
Pre-operative CT scan showing expansive and deforming growth on the face.

**Figure 2:**
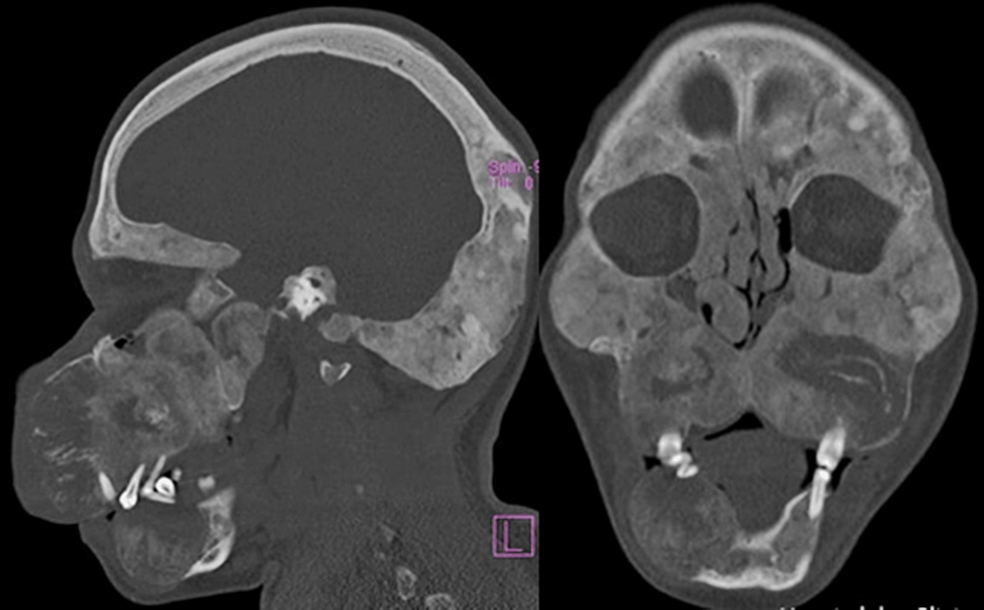
Diffuse craniofacial thickening, with expansive lytic lesions with a ground-glass pattern.

The tomographic examination revealed the presence of diffuse craniofacial thickening, with expansive lytic lesions in the bone marrow with a ground-glass pattern, associated with discrete cortical thinning, with emphasis on the expansive effect and regional deformity, notably in the maxilla and the parasymphysis region and mandibular body on the right side (Figures 1, 2).

Given the history, clinical and imaging aspects, a diagnostic hypothesis of recurrent growth of fibrous dysplasia and consequent surgical planning were established.

**Figure 3:**
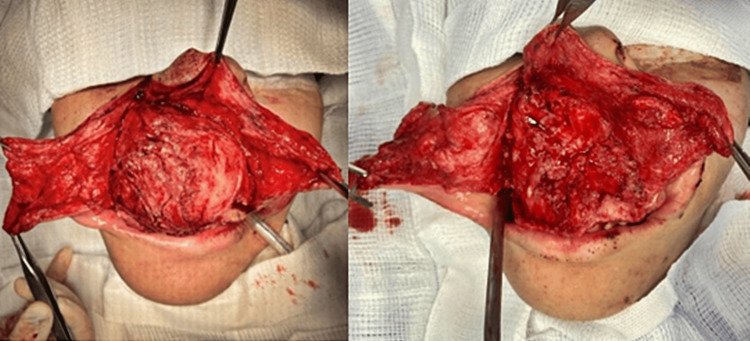
Weber-Ferguson approach and resection of the lesion with bone plasty.

As a result of the injuries, the patient presented limited mouth opening and was classified IV on the Mallampati airway assessment scale, requiring orotracheal intubation with the aid of a fiberscope. After induction of general anesthesia, the approach consisted of the Weber-Ferguson surgical approach for broad visualization and partial resection of the maxillary lesion initially with a chisel and hammer and later with rotary instruments in order to obtain bone plasty, with correction of spicules and irregularities (Figure 3) for the then closing using 4-0 vicryl (Ethicon, Inc., NJ, USA) and 5-0 nylon (AD Surgical, CA, USA).

**Figure 4:**
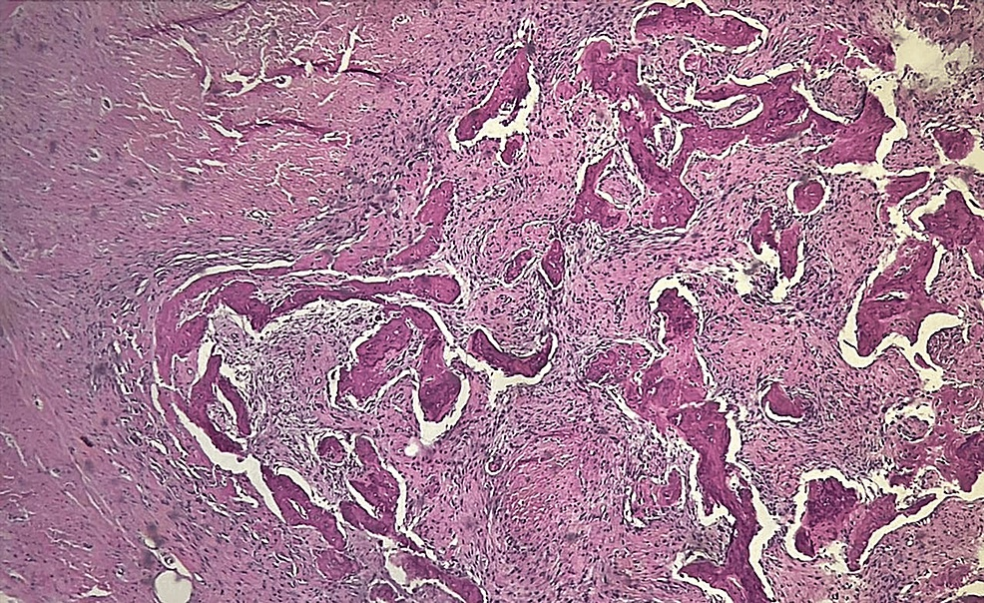
Cellular fibrous stroma and irregular bone trabeculae, hematoxylin and eosin stain, original magnification 100x.

Subsequently, the patient was sent to the intensive care unit (ICU) to ensure airway safety, being discharged after 12 hours of monitoring. The anatomopathological analysis revealed cellular fibrous stroma and irregular bone trabeculae, compatible with fibrous dysplasia (Figure 4).

**Figure 5:**
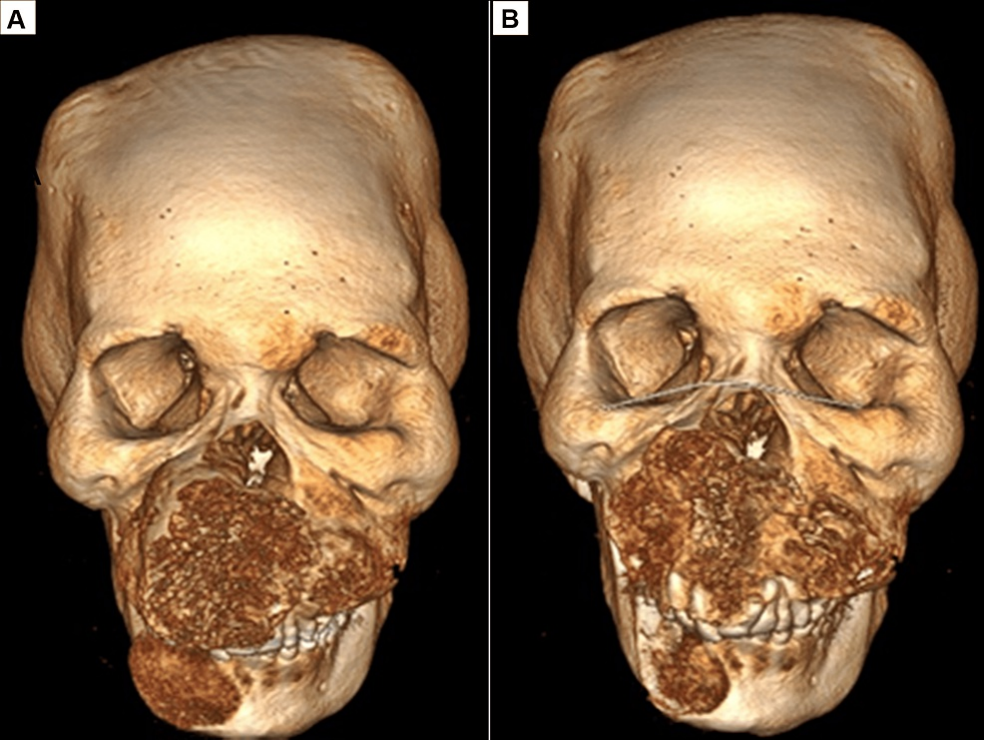
(A) Preoperative tomography. (B) Postoperative tomography.

**Figure 6:**
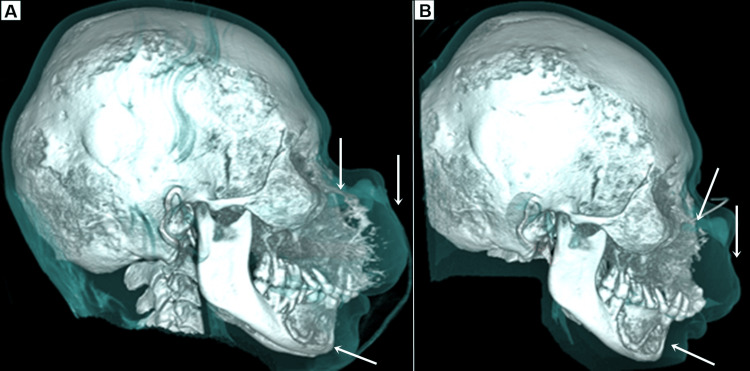
(A) Preoperative tomography showing maxillary and mandibular lesions with repercussions on soft tissues (arrows). (B) Postoperative tomography showing partial resection of the lesions with repercussions on soft tissues (arrows).

Currently, the patient is undergoing a three-month follow-up, with satisfactory evolution of the aesthetic and functional complaints, with complete respiratory recovery and will continue in follow-up given the alarming history of recurrences (Figures 5, 6).

## Discussion

The term fibro-osseous injury is used to designate a wide group of pathologies that affect hard tissue and affect skeletal architecture [6]. The etiology is variable and the pathogenesis is uncertain, with clinical, radiographic and histopathological characteristics that vary from developmental anomalies, hamartoma, and reactive and dysplastic lesions to benign neoplasia [6].

Within this group is fibrous dysplasia, which statistically represents 2% of bone tumors and 7% of benign bone tumors [3]. This condition results from a postzygotic mutation that generates replacements of amino acids, such as cysteine and histidine, in the genomic DNA in osteoblastic cells with another amino acid, arginine [6].

It is a developmental anomaly that interferes with bone maturation and remodeling, manifesting itself through the replacement of the normal bone apparatus by fibrous connective tissue with indefinite amounts of mineralization and calcification, generating a weaker structure [2-4,6,7].

Fibro-osseous lesions can be found in any part of the skeleton, however, they are more common in the craniofacial region and proximal femurs, and can manifest important clinical sequelae in these situations [2].

Based on this principle, it is important to emphasize that the complications of this pathology are strongly associated with the possibility of hearing loss due to dysplastic extension to the temporal bone, which is normally mild and non-progressive. As well as loss of vision, which is a serious but rare complication that can arise due to deformation of the optic canals [2,3].

In addition to these, other possible sequelae of craniofacial fibrous dysplasia include nasal congestion, hyposmia and malocclusion [2]. When relating to the above case, fortunately, no visual or auditory impairments were diagnosed, however, it clinically exemplifies the possible repercussions on the respiratory system in the face of hyposmia and respiratory difficulty, as well as malocclusion due to facial deformity and tooth mobility.

Another problem linked to bone fragility is redirected to the manifestation of dysplasia in the lower limbs, which becomes a source of morbidity and compromise in the face of fractures, which normally occur between six and ten years, before reaching adulthood, precociously impairing ambulation [2]. This circumstance is manifested in the aforementioned case in the report of eight fractures of the lower limbs, the first one was at the age of seven, which occurred without significant impact, and the current need to walk with the aid of a crutch.

These complications are consistent with the clinical presentation of fibrous dysplasia, which varies widely based on the location and amount of bone affected, and can present in two patterns: monostotic, when it involves a single bone with or without adjacent structures, and polyostotic when there is involvement of multiple bones, as in the case presented [2-4,7].

It is estimated that monostotic is the most common one, however, there is a lack of reliable epidemiological data due to the poor characterization of fibro-osseous lesions, which normally require biopsy and molecular testing to obtain a reliable diagnosis [2].

This classification is closely associated with the moment in which the mutation occurs, as it will define the extent and severity of bone involvement, as well as the presence of abnormalities [6]. If it occurs in a skeletal progenitor cell at later times in embryonic development, the mutant cell will be expressed in the skeletal formation, causing multiple bone involvement, that is, polyostotic fibrous dysplasia [6]. If the mutation occurs postnatally, only one bone will be affected, defining monostotic fibrous dysplasia [6].

In addition, in cases where the mutation reaches undifferentiated stem cells during the beginning of embryological life, the consequences will be expressed in osteoblasts, melanocytes and the endocrine system [6]. The latter justifies hormonal abnormalities and café-au-lait skin pigmentation, characteristic of McCune-Albright syndrome, a condition present in the case presented.

McCune-Albright syndrome, however, is a rare pathology with notable complexity, developed by somatic gain-of-function mutations in the GNAS gene, and it is believed that these changes are postzygotic, attributing characteristics sustained by the absence of vertical transmission. of the disease [1,8]. This condition was historically characterized by the union of polyostotic fibrous dysplasia, café-au-lait skin pigmentation and precocious puberty [1-3]. However, associations with the classic triad were later observed, mainly endocrinopathies, such as hyperthyroidism, excessive growth hormone, renal phosphate wasting with or without rickets/osteomalacia and Cushing's syndrome [1]. The classic manifestations of this triad undoubtedly make up the patient's clinical picture, who, in addition to pigmentation and fibrous dysplasia, has a history of menarche at six years of age, confirming precocious puberty.

Signs of fibrous dysplasia usually appear during childhood or adolescence, but the condition can remain asymptomatic and be diagnosed through routine imaging tests [3]. However, given possible complications and sequelae, such as facial asymmetries and deformities, rapid and accurate diagnosis are essential, which is obtained by combining clinical, imaging and histopathological examination [3].

Fibrous dysplasia presents, tomographically, with a “ground glass” appearance with well-defined edges, and microscopically, as discontinuous networks of bone trabeculae, suggesting aberrant osteoblastic activity with an irregular shape, resembling “Chinese characters” [2]. Both characteristics agree with the imaging and microscopic findings of the reported case.

The management of fibrous dysplasia can be done conservatively through observation and follow-up and surgical therapy through osteoplasty [5]. The therapeutic option through medication has been highlighted as a possibility, but it is still the subject of study, with a lack of sufficient evidence to justify the approach [5].

Therefore, medicine treatment includes the use of bisphosphonates, which are antiresorptive agents with the ability to increase bone remodeling and osteoclastogenesis of fibro-osseous tissue [2,5]. This class has been extensively studied in fibrous dysplasia and yet has no specific role in the clinical management of the condition [2]. Based on this, studies have shown that patients who used bisphosphonates reported a decrease in pain, as well as an improvement in markers of bone tissue renewal and a decrease in radiolucency on control radiographs in fibrous dysplasia in the femoral cortices [2]. Despite this, retrospective analyses did not verify associations between the use of bisphosphonates and craniofacial deformities, leading to the conclusion that the findings do not support the use of bisphosphonates in the prevention, expansion or improvement of mechanical properties of fibrous dysplasia [2,5].

Another medicine that participates in dysplasia therapy is denosumab, a monoclonal antibody with a promising proposal for osteoporosis and complications of skeletal tumors [2]. The association between this medication and dysplasia emerged after reports of its efficiency in the treatment of giant cell tumors, which in turn share histological characteristics with fibrous dysplasia [2]. From this, some studies were carried out with results that showed rapid improvement in pain and markers of bone remodeling, as well as an evident reduction in the expansion of lesions [2]. However, after discontinuation, there was a rapid rebound in bone resorption markers, which generated severe hypercalcemia, a complication associated with the use of denosumab in several cases [2]. Thus, current evidence points to the potential capacity for the treatment of fibrous dysplasia, however, there is concern and uncertainty regarding the evolution after discontinuation of the drug and use should be limited [2].

When considering the surgical approach, the treatment of fibrous dysplasia is challenging and mainly indicated to repair functional impairments, such as compression of nerve structures, malocclusion, and correct aesthetic deformities [2,3,5]. Typically, the approach occurs after puberty, due to the possibility of remission or stagnation of the fibro-osseous lesion [2,3,5].

An alarming factor associated with the fibrous dysplasia approach is associated with the tendency for growth to recur after subtotal resections, becoming an important limitation that generates “suboptimal” results [2]. The idea becomes even more worrying given studies that indicate new growth in 68% of cases of craniofacial resections, more frequent in conservative recontouring approaches in patients with excess growth hormone [2]. In association with this characteristic and the case presented, it becomes obvious to understand the potential chance of recurrence, since the patient underwent four approaches with the aim of partial resection and osteoplasty of the fibrous dysplasia in an interval of five years, even in the absence of growth hormone decompensation.

Despite the limitations, surgical intervention should be performed to correct functional impairments to obtain better symmetry and facial contour [2,3,5]. Several surgical approaches have been proposed for treatment according to the size and location of the lesions, including, the most common for the middle third are the buccogingival approach, coronal approach, Weber-Ferguson and degloving of the middle third [7]. In this specific case, given the extensive lesion in the maxilla, we opted to use the Weber-Ferguson method to obtain satisfactory access to the lesion aiming to carry out proper resection, while the smaller mandibular lesion was calmly resected using the Modified Newman flap.

Furthermore, it is essential to reinforce that the technique must be individualized [2] and involve total resection, indicated for isolated lesions or partial resection with conservative facial realignment [3,7]. In the case presented, complete resection is unfeasible given the involvement of multiple bones and important structures, opting for partial resection and realignment or plasty of the lesions to obtain greater permeability of the nostrils and a satisfactory facial contour.

The importance of obtaining satisfactory results becomes even more fundamental given studies that demonstrate that self-perception of the manifestations of fibrous dysplasia is associated with impairments in quality of life [4].

Currently, the patient is undergoing a 90-day follow-up, with satisfactory evolution of his functional and aesthetic condition and will continue to be monitored in accordance with the monthly clinical and semi-annual tomographic reassessment protocol, as proposed by Kang [7].

## Conclusions

Given the clinical case presented, fibrous dysplasia is a pathology of varying degrees of severity, which may have important functional and aesthetic implications with consequences for quality of life and psychosocial factors. Therefore, when diagnosing this condition, it is necessary to analyze the extent of the impairments and consider the best form of treatment for the patient. Thus, despite the chances of recurrence in some cases, functional and aesthetic complaints can be repaired through a surgical technique with partial resection of the lesion and facial realignment, resulting in an improvement in the patient's quality of life.
